# Supplementary material for: Patients’ perceptions regarding multidrug-resistant tuberculosis and barriers to seeking care in a priority city in Brazil during COVID-19 pandemic: A qualitative study
Source: PLoS One. 2021 Apr 9;16(4):e0249822. doi: 10.1371/journal.pone.0249822 (PMC8034748; doi:10.1371/journal.pone.0249822)
Supplement: S1 File — (PDF) [file pone.0249822.s001.pdf]

Date: _____/_____/_____

ID code: _______________

SEMI-STRUCTURED INTERVIEW SCRIPT

SEMI-STRUCTURED INTERVIEW SCRIPT FOR PEOPLE WITH MULTIDRUG-RESISTANT TUBERCULOSIS

**1.** Is this the first time that you have been treated for tuberculosis?
**2.** In your opinion, does multidrug-resistant tuberculosis have a cure?
**3.** How has been your experience of dealing with multidrug-resistant tuberculosis?
**4.** What were the difficulties in your treatment experience since the moment you were diagnosed?
**5.** In your personal experience, what could be the cause of your illness?
**6.** Have you ever abandoned treatment? (If yes, ask to the participant why!)
**7.** Do you have support from your family and friends? Are you living with their?
**8.** How has your experience with health services been since you became ill?
**9.** Have you received any benefits since you became ill?
**10.** How long have you been treating? Did the medication have any side effects? (If yes, ask to the participant which side effects!)
**11.** What do you think is most positive about the treatment? Is there anything negative?
**12.** How has it been the treatment of multidrug-resistant tuberculosis during the COVID-19 pandemic?
