# Supplementary material for: Patients’ perceptions regarding multidrug-resistant tuberculosis and barriers to seeking care in a priority city in Brazil during COVID-19 pandemic: A qualitative study
Source: PLoS One. 2021 Apr 9;16(4):e0249822. doi: 10.1371/journal.pone.0249822 (PMC8034748; doi:10.1371/journal.pone.0249822)
Supplement: S2 File — (PDF) [file pone.0249822.s002.pdf]

Consolidated criteria for reporting qualitative studies (COREQ): 32-item checklist

Developed from:

Tong A, Sainsbury P, Craig J. Consolidated criteria for reporting qualitative research (COREQ): a 32-item checklist for interviews and focus groups. *International Journal for Quality in Health Care*. 2007. Volume 19, Number 6: pp. 349 – 357

A checklist of items that should be included in reports of qualitative research.

YOU MUST PROVIDE A RESPONSE FOR ALL ITEMS. ENTER N/A IF NOT APPLICABLE

No. Item	Guide questions/description	Reported on Page #
**Domain 1: Research team and reflexivity**		
Personal Characteristics		
1. Interviewer/facilitator	Which author/s conducted the interview or focus group?	12
2. Credentials	What were the researcher's credentials? E.g. PhD, MD	12
3. Occupation	What was their occupation at the time of the study?	12
4. Gender	Was the researcher male or female?	12
5. Experience and training	What experience or training did the researcher have?	12
Relationship with participants		
6. Relationship established	Was a relationship established prior to study commencement?	N/A
7. Participant knowledge of the interviewer	What did the participants know about the researcher? e.g. personal goals, reasons for doing the research	13
8. Interviewer characteristics	What characteristics were reported about the interviewer/facilitator? e.g. Bias, assumptions, reasons and interests in the research topic	12
**Domain 2: Study design**		
Theoretical framework		
9. Methodological orientation and Theory	What methodological orientation was stated to underpin the study? e.g. grounded theory, discourse analysis, ethnography, phenomenology, content analysis	7-8

Participant selection		
10. Sampling	How were participants selected? e.g. purposive, convenience, consecutive, snowball	10
11. Method of approach	How were participants approached? e.g. face-to-face, telephone, mail, email	10
12. Sample size	How many participants were in the study?	10
13. Non-participation	How many people refused to participate or dropped out? Reasons?	10
Setting		
14. Setting of data collection	Where was the data collected? e.g. home, clinic, workplace	12
15. Presence of non-participants	Was anyone else present besides the participants and researchers?	N/A
16. Description of sample	What are the important characteristics of the sample? e.g. demographic data, date	14-16
Data collection		
17. Interview guide	Were questions, prompts, guides provided by the authors? Was it pilot tested?	11
18. Repeat interviews	Were repeat inter views carried out? If yes, how many?	N/A
19. Audio/visual recording	Did the research use audio or visual recording to collect the data?	12
20. Field notes	Were field notes made during and/or after the inter view or focus group?	12
21. Duration	What was the duration of the inter views or focus group?	12
22. Data saturation	Was data saturation discussed?	10
23. Transcripts returned	Were transcripts returned to participants for comment and/or correction?	N/A
**Domain 3: Analysis and findings**		
Data analysis		
24. Number of data coders	How many data coders coded the data?	16
25. Description of the coding tree	Did authors provide a description of the coding tree?	N/A
26. Derivation of themes	Were themes identified in advance or derived from the data?	16
27. Software	What software, if applicable, was used to manage the data?	12
28. Participant checking	Did participants provide feedback on the findings?	N/A
Reporting		
29. Quotations presented	Were participant quotations presented to illustrate the themes/findings? Was each quotation identified? e.g. participant number	16-21

30. Data and findings consistent	Was there consistency between the data presented and the findings?	23-29
31. Clarity of major themes	Were major themes clearly presented in the findings?	16-23
32. Clarity of minor themes	Is there a description of diverse cases or discussion of minor themes?	23-29

Once you have completed this checklist, please save a copy and upload it as part of your submission.

When requested to do so as part of the upload process, please select the file type: *Checklist*.

You will NOT be able to proceed with submission unless the checklist has been uploaded.

Please DO NOT include this checklist as part of the main manuscript document. It must be uploaded as a separate file.
